# Virtual Screening for Organic Solar Cells and Light Emitting Diodes

**DOI:** 10.1002/advs.202200825

**Published:** 2022-04-22

**Authors:** Nancy C. Forero‐Martinez, Kun‐Han Lin, Kurt Kremer, Denis Andrienko

**Affiliations:** ^1^ Max Planck Institute for Polymer Research Ackermannweg 10 Mainz 55128 Germany

**Keywords:** chemical design, light emitting diodes, organic electronics, solar cells

## Abstract

The field of organic semiconductors is multifaceted and the potentially suitable molecular compounds are very diverse. Representative examples include discotic liquid crystals, dye‐sensitized solar cells, conjugated polymers, and graphene‐based low‐dimensional materials. This huge variety not only represents enormous challenges for synthesis but also for theory, which aims at a comprehensive understanding and structuring of the plethora of possible compounds. Eventually computational methods should point to new, better materials, which have not yet been synthesized. In this perspective, it is shown that the answer to this question rests upon the delicate balance between computational efficiency and accuracy of the methods used in the virtual screening. To illustrate the fundamentals of virtual screening, chemical design of non‐fullerene acceptors, thermally activated delayed fluorescence emitters, and nanographenes are discussed.

## Graphical abstract

Computational methods which point to new, better organic semiconductors require both diverse molecular databases and efficient and accurate methods used in the virtual screening. To illustrate the screening workflow, chemical design of non‐fullerene acceptors, thermally activated delayed fluorescence emitters, and nanographenes are discussed.

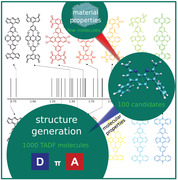

## 1 Introduction

Carbon is not only fundamental for life on our planet but also plays an important role in shaping the human economy, technology and society. Arguably, the key property that makes carbon a versatile building block is its ability to form up to four covalent bonds, whose energy is approximately two orders of magnitude larger than *k*_B_ *T* with *k*_B_ the Boltzmann constant and *T* the (room) temperature. As a result, carbon forms a wide variety of stable structures, such as graphite, diamond, carbon nanotubes, and fullerenes as well as all commodity polymers.

Out of this wealth of structures, conjugated molecules are of particular interest: the aromaticity of benzene, where delocalized π orbitals contribute to both stability and spectroscopic activity^[^ ^1^ ^]^ is the representative example here. Benzene's stability and rich electronic structure enable the synthesis of other aromatic compounds, such as polycyclic aromatic hydrocarbons (PAHs)^[^ ^2^ ^]^ and graphene.^[^ ^3^ ^]^ Conductive polymers represent another important type of conjugated systems. The discovery of polyacetylene, the first electrically conductive conjugated polymer,^[^ ^4^ ^]^ opened perspectives for novel printed electronic devices.^[^ ^5^ ^]^ The rapid expansion of the field of organic semiconductors was soon acknowledged by the Nobel Prize in chemistry, awarded jointly to Alan J. Heeger, Alan G. MacDiarmid, and Hideki Shirakawa “for the discovery and development of conductive polymers.”^[^ ^6^, ^7^, ^8^ ^]^

The current progress in the field is largely driven by the expansion of the database of conjugated molecules, which are constantly scrutinized for applications in solar cells, field effect transistors, light emitting diodes, and electrodes. The emerging trend is a rational, application‐driven design of this database. Here, computational high‐throughput screening methods are starting to guide the discovery of new materials. First, by helping to pre‐screen virtual databases for structures with predefined properties. Second, by establishing clear structure‐property relationships.

The term “structure‐property relationship” is, to a certain extent, self‐explainable: it refers to a link between the chemical structure and the physical property of an organic semiconducting material. A representative example of a structure‐property relationship is a link between the molecular structure and the ionization energy (IE) or electron affinity (EA) of a film of such molecules, which are the relevant energy levels for transport of holes and electrons, respectively.

Predicting material properties, such as IE or EA, has two interrelated aspects: computational cost and accuracy. Hypothetically, we could provide an exact distribution of IE or EA (density of states) by solving the Schrödinger equation for the entire film, provided that local packing and morphology are exactly known. This is practically impossible: first, the material morphology depends on the processing conditions, which are difficult to mimic in computer simulations; second, we are limited by computational resources and prohibitive scaling of computational cost with the system size. Therefore, we have to resort to approximate models. A perturbative scheme, for example, treats one molecule quantum mechanically, while the environment is treated classically, using, for example, a polarizable force‐field.^[^ ^9^, ^10^ ^]^ In other words, we are balancing the computational cost and accuracy of the prediction by devising a simplified model with a suitable computational overhead.

**Figure 1:**
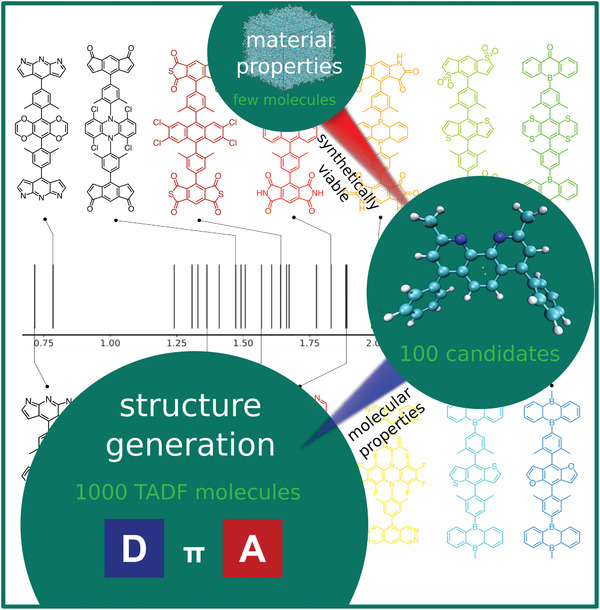
Funnel‐like virtual screening workflow. The database of compounds is generated according to a predefined molecular template, for example, the donor–bridge–acceptor architecture. Computed molecular properties are then used to select a subset of molecules, for which computationally‐demanding multiscale simulations are further performed.

In fact, it is useful to have a hierarchy of such models, as illustrated in **Figure** **1**. The less accurate models prefilter the database of compounds, and the computationally demanding ones refine the prefiltered database. For example, the first approximation of IE of a solid film can be obtained by calculating the energy of the highest molecular orbital of a molecule. This is a crude approximation, for several reasons: Koopman's theorem, that is, IE is equal to the negative of the highest occupied molecular orbital (HOMO) energy, does not hold for approximate density functional theory (DFT) functionals,^[^ ^11^, ^12^ ^]^ as well as we are approximating the solid state with the gas‐phase ionization energy. As a consequence, first prescreening should allow for a larger range of HOMO variations, say ±1 eV, which would account for the error introduced by the neglect of stabilization energies in a dielectric media. In the next step, energies of a cation and an anion can be refined, for example using the omega‐tuning procedure^[^ ^13^ ^]^ and implicit solvent. Finally, for a few selected compounds, one can parameterize the atomistic force‐field, simulate atomistically‐resolved morphologies, and perform perturbative calculations of IE and EA.

The funnel‐like approach described above is still impractical in most cases, because the number of organic molecules in virtual databases exceeds—by far—available computational resources. To further reduce the size of the trial database, we need to select a certain class of chemical structures, which is suitable for the properties that we need for our calculations. For example, one can prescreen only donors in the donor–acceptor type molecules, in which the acceptor block is fixed and which then sets the ionization energy.

Predefining the prototype of the molecular architecture requires a fairly deep understanding of the problem. In this respect, machine learning (ML) techniques are becoming more and more popular.^[^ ^14^, ^15^, ^16^ ^]^ In fact, ML models have already defeated the world champions in chess and go^[^ ^17^ ^]^ as well as brought us forward in predicting protein folding^[^ ^18^ ^]^ and even electron densities.^[^ ^19^ ^]^ It remains to be seen how successful these methods will be in the coming years in the present context.

In the next sections we illustrate computer‐aided design of thermally activated delayed fluorescent dyes, non‐fullerene acceptors, and nanographenes.

## 2 Design of Thermally‐Activated Delayed Fluorescence Emitters

**Figure 2:**
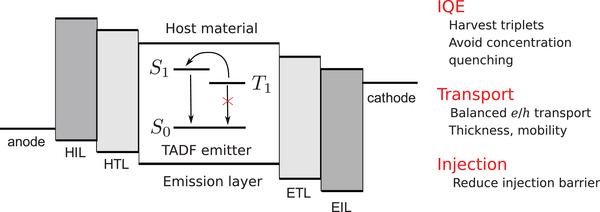
The device architecture of state‐of‐the‐art OLED devices. Each layer has its own function to achieve high device efficiency: Electron/hole injection layer is to reduce the injection barrier of electron/hole from the electrode to the emission layer; Electron/hole transport layer is to balance the electron and hole transport and to restrict the electron/hole recombination at the emission layer; Emission layer is to convert injected electrons and holes into photons of a specific wavelength. It is often a guest–host structure, where organic emitters (guest) are doped into an inactive host material to prevent concentration quenching.

An interesting example of an application‐driven molecular design is the optimization of compounds in an organic light emitting diode (OLED).^[^ ^20^, ^21^ ^]^ A modern state‐of‐the‐art OLED in general possesses a multilayer device architecture, which is composed of two electrodes, electron/hole injection layers,^[^ ^22^ ^]^ electron/hole transport layers and emission layers, as shown in **Figure** **2**. Injection of electrons and holes in such a device leads to both singlet (25%) and triplet (75%) excited states. To reach 100% internal quantum efficiency (IQE), several design strategies of emitters have been proposed to harvest triplet states.^[^ ^23^, ^24^, ^25^, ^26^ ^]^ Here, we focus on a class of emitters called thermally activated delayed fluorescence (TADF) emitters.^[^ ^27^ ^]^

A TADF emitter has relatively small, on the order of 0.01 eV, energy gap between its singlet *S*_1_ and triplet *T*_1_ states. The reverse intersystem crossing (rISC) can therefore occur at room temperature. As a result, OLEDs with a TADF emitter can harvest triplet states.

**Figure 3:**
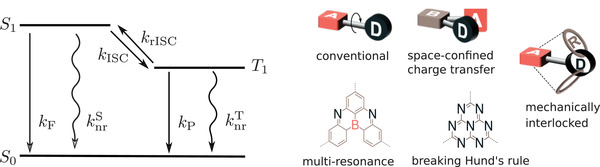
a) Key processes taking place in a TADF emitter. *k*_F_ is the fluorescence rate, *k*_nr_ is the nonradiative decay rate, *k*_(r)ISC_ is the (reverse) intersystem crossing rate, and *k*_P_ is the phosphorescence rate. b) Design strategies for efficient TADF emitter.

Apart from rISC, OLED efficiency depends on the fluorescence rate, non‐radiative decay rate, and the phosphorescence rate. These processes are illustrated in **Figure** **3**. In general, the requirements of a high‐performance TADF emitter are high *k*_F_, high *k*_rISC_, low *k*_P_, and low *k*_nr_. Therefore, it is important to develop models that help to evaluate these rates from the underlying molecular structures, providing insights for the molecular design.^[^ ^28^ ^]^ For instance, the fluorescence rate can be estimated using the Einstein's spontaneous emission equation,^[^ ^29^ ^]^ $k_{F}=f_{S_{0}S_{1}}\Delta E_{S_{0}S_{1}}^{2}/1.499\;{\text{cm}}^{-2}\;s$, where $f_{S_{0}S_{1}}$ is the oscillator strength and $E_{S_{0}S_{1}}^{2}$ (in cm^−1^ ) is the energy difference between the *S*_1_ and *S*_0_ states. For TADF emitters with a fixed target color, high oscillator strength of the emitter is therefore a relevant figure of merit.

Similarly, the phosphorescence rate can be estimated using the same form as the fluorescence rate,^[^ ^30^ ^]^ $k_{P}=f_{S_{0}T_{1}}\Delta E_{S_{0}T_{1}}^{2}/1.499\;{\text{cm}}^{-2}\;s$, where $\Delta E_{S_{0}T_{1}}^{2}$ is the energy difference between *T*_1_ and *S*_0_ states. Note that the oscillator strength $f_{S_{0}T_{1}}$ between pure *S*_0_ and *T*_1_ states is zero. Therefore, allowing *S*_0_/*T*_1_ to gain admixtures from states with triplet/singlet states in the framework of perturbation theory is crucial to get nonzero $f_{S_{0}T_{1}}$.

For the (reverse) intersystem crossing rate a computationally efficient approach is to adopt the Marcus rate expression^[^ ^31^ ^]^

(1) $$\begin{array}{c} k_{\text{rISC}}=\frac{{\left|H_{\text{SO}}^{S_{1}T_{1}}\right|}^{2}}{\hbar }\sqrt{\frac{\pi }{\lambda k_{B}T}}\exp\left[-\frac{{\left(\Delta E_{\text{ST}}+\lambda \right)}^{2}}{4\lambda k_{B}T}\right]\; \end{array}$$

Here, three parameters determine the transition rate between the *S*_1_ and *T*_1_: the spin‐orbit coupling $H_{\text{SO}}^{S_{1}T_{1}}$, the reorganization energy λ and the energy difference between the two states, Δ*E*_ST_ (the sign is opposite for rISC and ISC). Overall, large spin‐orbit coupling and small Δ*E*_ST_ + λ is favorable.

Finally, within the Franck–Condon approximation, the rate of nonradiative decay via internal conversion (IC) from *S*_1_ to *S*_0_ can be written as^[^ ^32^ ^]^

(2) $$\begin{array}{c} k_{\text{nr}}=\frac{2\pi }{\hbar }\sum_{lk}\frac{R_{lk}^{f}}{Z_{i\nu }}\sum_{\nu_{i}\nu_{f}}e^{-\beta E_{i\nu_{i}}}P_{lk}^{f}\delta \left(E_{if}+E_{i\nu_{i}}-E_{f\nu_{f}}\right) \end{array}$$

where *Z*_*iv*_ is the partition function, $R_{lk}^{f}=\langle \Phi_{f}|{\hat{P}}_{fl}|\Phi_{i}\rangle \langle \Phi_{i}|{\hat{P}}_{fk}\left|\Phi_{f}\right\rangle$, ${\hat{P}}_{fl}$ is the mass weighted normal momentum operator, ${\hat{P}}_{fl}=-i\hbar \partial /\partial Q_{fl}$ is the *l*th normal mode coordinate of the final state, $P_{lk}^{f}=\langle \Theta_{f\nu_{f}}|{\hat{P}}_{fl}|\Theta_{i\nu_{i}}\rangle \langle \Theta_{i\nu_{i}}|{\hat{P}}_{fk}\left|\Theta_{f\nu_{f}}\right\rangle$, *Q*_*fl*_ is the *l*th normal mode coordinate of the final state, Φ_*i*/*f*_ and Θ_*i*/*f*_ are the electronic and vibrational mode wavefunctions of the final states, respectively,

The evaluation of *k*_nr_ using Equation (2) involves computations of non‐adiabatic coupling matrix elements, which is computationally demanding. Alternatively, the cost‐efficient descriptor, reorganization energy $\lambda_{S_{1}S_{0}}$, can be used to measure the feasibility of IC between *S*_1_ and *S*_0_, where large reorganization energy generally leads to large nonradiative decay rates.^[^ ^33^ ^]^

From the computational materials science perspective, many parameters appearing in the rate equations can be obtained from first principles, providing a direct link between the molecular conformation and the rate. Indeed, a number of molecular design strategies which help to tune the ingredients of these rates have been proposed, as summarized in Figure 3.

To begin with, the conventional TADF design relies on the reduction Δ*E*_ST_ due to the decrease of the spatial overlap of the HOMO and the lowest unoccupied molecular orbital (LUMO). In a donor–acceptor (D–A) TADF molecule, this is achieved by making the donor and the acceptor orthogonal to each other.^[^ ^27^ ^]^ Despite the favorable small Δ*E*_ST_, a perfect orthogonal D–A compound has $f_{S_{1}S_{0}}=0$ due to the CT character of *S*_1_, leading to *k*_F_ = 0. The nonradiative condition is resolved with the help of vibrational motions and the conformational disorder in the solid state, bringing the molecule away from orthogonality.^[^ ^34^ ^]^ In other words, a flexible dihedral angle between donor and acceptor (ϕ_DA_) is responsible for broad emission spectra and large *k*_IC_.^[^ ^28^ ^]^ Therefore, tailoring the molecular design and the choice of the host material is crucial to reach the sophisticated balance between rates of different processes.^[^ ^35^ ^]^

Space‐confined charge‐transfer (SCCT) is another concept that can be used to boost the efficiency of a TADF emitter. Here, a molecule consists of cofacially arranged donors and acceptors that are connected by a rigid linker.^[^ ^36^ ^]^ In fact, two design philosophies can lead to the SCCT molecular architecture. From the perspective of refining the conventional TADF emitters, Chen et al. showed that the noncovalent interaction between the donor and the acceptor in a sterically congested molecular geometry lead to the hybridization of the CT and LE states in *S*_1_ and *T*_1_. This enhances the $f_{S_{0}S_{1}}$ and $H_{\text{SO}}^{S_{1}T_{1}}$, increasing *k*_F_ and *k*_(r)ISC_. In addition, the ortho substitution of the donor and acceptor gives it a “locked” character, resulting in small $\lambda_{S_{1}S_{0}}$ and therefore slow *k*_nr_.

From the perspective of improving the conventional TADF exciplex, Tang et al. arrived at the same idea of SCCT.^[^ ^37^ ^]^ The exciplex emission is dominated by the distance between the donor and the acceptor compounds, where large donor–acceptor separation leads to negligible exciplex emission. The attempt of confining the D–A distance by linking the donor and acceptor with a spacer (D–σ–A), the so‐called through‐space charge‐transfer (TSCT), was not very successful. It turned out that the control of the relative orientation (coupling) between the donor and the acceptor is essential, and can be achieved via the SCCT design.^[^ ^38^ ^]^

Another strategy utilizing the intramolecular noncovalent interactions is the mechanically interlocked molecular design. The idea is to modify the conformational dynamics and hence the rate of critical photophysical process. Rajamalli et al. demonstrated that the carbazole–benzophenone‐based rotaxanes exhibit better performance as compared to their noninterlocked counterpart.^[^ ^39^ ^]^ In this particular case, the introduction of the mechanical bond leads to increased PL quantum yield and photostability, reduced Δ*E*_ST_, shallower HOMO, and red‐shift in the emission spectrum.

One of the drawbacks of conventional D–A TADF emitters is that the desirable small Δ*E*_ST_ correlates with small $f_{S_{1}S_{0}}$. To overcome this limitation, the multiresonant (MR) TADF emitter has been proposed.^[^ ^40^, ^41^ ^]^ MR‐TADF has a planar fused aromatic ring with electron donating atoms and electron deficient atoms arranged in para positions to each other, as shown in Figure 3. Pershin et al. showed that the MR‐TADF emitters can exhibit both small Δ*E*_ST_ and high $f_{S_{1}S_{0}}$,^[^ ^42^ ^]^ which opens the possibility to increase the device performance. However, the MR‐TADF emitters are rare, and most of them are nanographenes doped with both donor and acceptor atoms, such as O, B, and N.^[^ ^41^ ^]^ Recently proposed DilCzMes4 is the first acceptor‐free MR‐TADF that contains only nitrogen as donor,^[^ ^43^ ^]^ showing an unexplored frontier in MR‐TADF design.

From the viewpoint of computational design, linear response time‐dependent (TD) DFT with popular functionals such as B3LYP, PBE0, or LC‐ωPBE significantly overestimates the Δ*E*_ST_ of MR‐TADF compounds. The poor prediction of TD‐DFT is ascribed to the lack of double excitations, since the methods that include double excitations such as SCS‐CC2 successfully predict Δ*E*_ST_.^[^ ^42^, ^44^ ^]^ SCS‐CC2 is a reliable but computationally demanding method, rendering it impractical for computational high‐throughput screening. Therefore, a reliable and cost‐effective method for prediction for MR‐TADF compounds is desirable, for example, the double‐hybrid functionals, as revealed in the recent benchmark study on Hund's‐rule‐violating molecules.^[^ ^45^ ^]^

Most compounds obey Hund's multiplicity rule, where their *T*_1_ states lie below their *S*_1_ states. Up to now, only few compounds violate Hund's rule,^[^ ^46^ ^]^ showing a negative Δ*E*_ST_. The existence of TADF compounds with negative Δ*E*_ST_ verifies the prediction by de Silva et al. based on a four‐state model.^[^ ^47^ ^]^ Despite the highly desirable inverted *S*_1_ and *T*_1_ states, only heptazine‐based compounds have been used so far in OLED devices.^[^ ^48^ ^]^ The design rule for novel Hund's‐rule‐violating molecules remains unclear. In addition, the prediction of negative Δ*E*_ST_, a phenomenon that can only be described beyond single excitations, is not achievable with TD‐DFT using common density functionals. Therefore, wavefunction methods including double excitations are usually required to obtain inverted *T*_1_ and *S*_1_.^[^ ^46^ ^]^ Fortunately, a recent benchmark study by Sancho‐Garcia et al. showed that TD‐DFT with double‐hybrid functionals can give the correct negative feature of Δ*E*_ST_.^[^ ^45^ ^]^ These methods can thus be used in computational high‐throughput screening to further increase the number of Hund's‐rule‐violating molecules.

**Figure 4:**
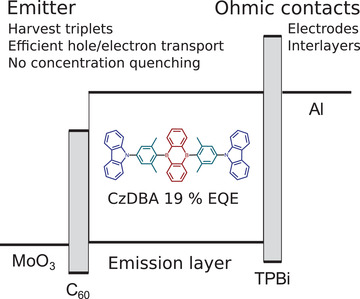
The device architecture of single‐layer OLED devices.

The discussion above is based on TADF emitters in multilayer OLEDs. A different strategy is to simplify the OLED architecture and use a single‐layer OLED,^[^ ^49^ ^]^ where the device is composed of only a pristine TADF film and electrodes, as shown in **Figure** **4**. The design strategy for this concept is far from trivial. In addition to triplet harvesting, a strategy of injecting electrons and holes into wide‐gap semiconductors is required^[^ ^50^ ^]^ as well as balanced electron and hole transport in a semiconducting film.

CzDBA is an example of such a compound.^[^ ^49^ ^]^ This compound is truly unique in a way of how many design principles it combines. First, its IE and EA lie within the recently‐identified “trap‐free” window,^[^ ^51^ ^]^ leading to a trap‐free transport. Second, high electron/hole mobility is achieved due to zero molecular dipole moment, resulting in small energetic disorder for electron and hole transport.^[^ ^52^, ^53^, ^54^, ^55^ ^]^ It is also a TADF emitter: it has small Δ*E*_ST_ (0.016 eV),^[^ ^56^ ^]^ achieved by making the donor and the acceptor orthogonal to each other via the m‐xylene bridge. Large $H_{\text{SO}}^{S_{1}T_{1}}$ is achieved by exhibiting large differences between excited‐state characters of *S*_1_ and triplet states lying close to *S*_1_.

In a solid film, it avoids, at least partially, concentration quenching. The latter is often caused by the formation of excimers in (nearly) cofacially arranged dimers (chromophores) with strong intermolecular interactions. In CzDBA this is resolved by introducing bulky groups (m‐xylene bridges) as shielding units. Similar strategies have also been used to design quenching‐resistant MR‐TADF emitter.^[^ ^57^ ^]^

**Figure 5:**
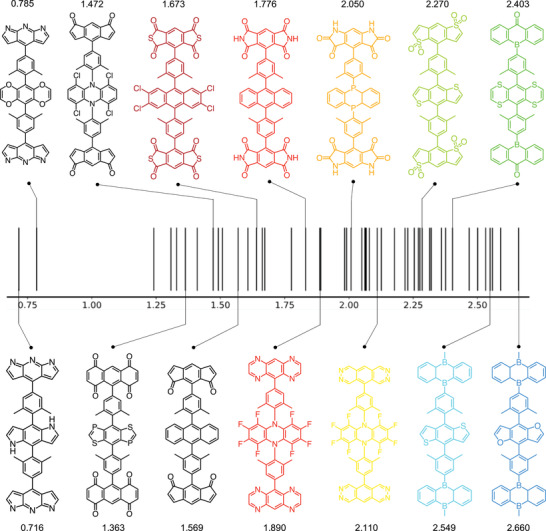
The estimated EL spectrum maximum (in eV) of 49 A–π–D–π–A candidates of single‐layer OLED emitters. The molecular structures of the 14 selected compounds are depicted. Reproduced with permission.^[^ ^56^ ^]^ Copyright 2021, Frontiers.

Searching for molecules simultaneously fulfilling all these requirements is challenging and virtual screening is a must. In our recent work, we constructed  1000 CzDBA‐like compounds (441 A–π–D–π–A and 504 D–π–A–π–D) with prescreened “trap‐free” donors and acceptors.^[^ ^56^ ^]^ Overall, we obtained ≈100 potential TADF emitters for single‐layer OLEDs with various EL spectrum maximum, as shown in **Figure** **5**, ranging from infrared (0.716 eV) to blue color (2.660 eV), which paves the way for future development of single‐layer OLED devices.

## 3 Non‐Fullerene Acceptors

Another representative example of virtual design of organic semiconductors is the development of the donor–acceptor combinations for organic solar cells (OSCs). The chemical design of OSC donors and acceptors was initially focusing mostly on the donors, where both small molecules and polymers were scrutinized^[^ ^58^, ^59^ ^]^ The acceptors were limited to a few structures, such as C60, C70,^[^ ^60^ ^]^ and their soluble version, PCBM.^[^ ^61^, ^62^ ^]^ It has soon been realized that the used acceptor molecules do not generate excitons and therefore free charges, because of their moderate light absorption.

The search of alternative, light‐absorbing, acceptors was not very successful for almost a decade, and the power conversion efficiencies (PCEs) of organic solar cells stagnated around 10%.^[^ ^64^ ^]^ Eventually, a novel class of molecules, coined as non‐fullerene acceptors (NFAs), have been designed, leading to a twofold increase of PCE of OSCs.^[^ ^65^, ^66^, ^67^, ^68^ ^]^ At present, OSCs based on small molecule non‐fullerene acceptors have power conversion efficiencies up to 17.4% for single junctions^[^ ^69^, ^70^, ^71^, ^72^ ^]^ and 18.6% for all‐organic solution‐processed tandem cells,^[^ ^73^, ^74^ ^]^ while fullerene‐based OSCs are only 10% efficient.

In an organic solar cell, a photogenerated exciton dissociates at the donor‐acceptor interface into an interfacial charge transfer state. The ionization energy or electron affinity energy offset at the heterojunction provides the driving force for the excited state dissociation that proceeds via a hole or an electron transfer. This offset should exceed a certain value, ≈0.5 eV, in order to enable efficient dissociation of the excited state.^[^ ^75^, ^76^, ^77^ ^]^ For the small optical gap materials, such as NFAs, only the ionization energy offsets are relevant, because of the fast energy transfer from the donor to acceptor.^[^ ^77^ ^]^

The interfacial CT state further dissociates into a pair of free charges—an endothermic process. The exact mechanism behind the driving force for this process is under debate.^[^ ^78^, ^79^, ^80^, ^81^, ^82^, ^83^ ^]^ It is one of the key processes in OSCs, since it determines, to a large extent, the open circuit voltage of organic heterojunctions.^[^ ^82^, ^84^, ^85^, ^86^ ^]^

The main difficulty in virtual screening of the donor/acceptor pairs is that any changes to their chemical structures affect simultaneously the open‐circuit voltage, the short‐circuit current, and the fill factor of the solar cell.^[^ ^87^, ^88^, ^89^, ^90^, ^91^, ^92^ ^]^ Without knowing how these changes correlate with each other, it is impossible to formulate clear molecular design rules. Some correlations can be established by incorporating, for example, electronic structure of the donor‐acceptor pair into the description, either phenomenologically,^[^ ^93^, ^94^ ^]^ or taking into account the underlying molecular architecture of the acceptor.^[^ ^63^ ^]^ For example, rigid elongated planar cores favor the formation of spatially extended, well‐ordered domains, about 10–30 nm in size.^[^ ^95^ ^]^ Rigid planar cores and large electronic couplings result in superior exciton diffusion lengths, up to 50 nm.^[^ ^96^ ^]^ As a result, the bulk heterojunction becomes more robust with respect to the domain size variation. Furthermore, electron affinities lower than −3 eV ensure trap‐free electron transport.^[^ ^51^, ^97^ ^]^

**Figure 6:**
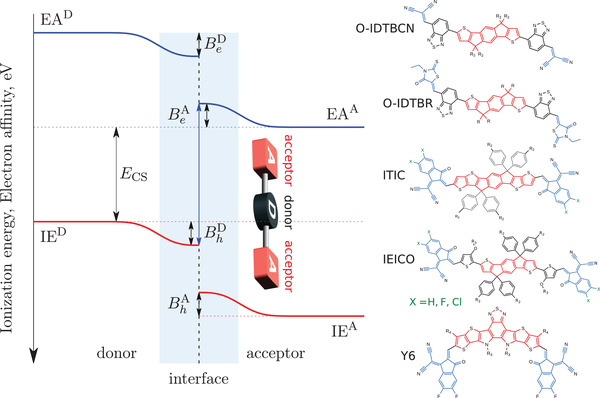
Sketch of the energy level diagram at the donor–acceptor interface illustrating the concepts of the energy level bending and (positive) interfacial bias potential. The electron is more stable in the phase with lower electron affinity (larger negative energy values) and the hole is more stable in the material with the higher ionization energy. The direction of the energy level bending corresponds to the A–D–A molecular architecture and long molecular axes oriented parallel to the donor‐acceptor interface. Positive interfacial bias destabilizes the charge transfer state, helping to dissociate it into the charge separated state. Chemical structures of typical NFA compounds illustrate the A–D–A molecular architecture. Adapted with permission.^[^ ^63^ ^]^ Copyright 2021, Wiley‐VCH.

In addition to these generic design rules, the electrostatic potential distribution at the donor–acceptor interface imposes additional constraints onto the molecular architecture of the acceptor. Donor–acceptor intermixing at the donor–acceptor interface leads to the electrostatic potential bending at the interface, as shown in **Figure** **6**.^[^ ^63^, ^98^, ^99^ ^]^ The resulting electrostatic potential destabilizes the charge transfer state, driving its dissociation into free charges. Potential bending in excess of 0.5 eV compensates the electron‐hole Coulomb binding energy, leading to barrier‐less dissociation of the CT state in free charges.^[^ ^63^, ^100^ ^]^ The energy level bending reduces the driving force required for hole transfer into the acceptor to the donor, leading to the formation of charge transfer states. As a result, 0.5 eV offset between ionization energies of the donor and acceptor is required for efficient hole transfer reactions.^[^ ^77^ ^]^

The energy level bending at the donor–acceptor interface can be traced backed to the molecular crystal field.^[^ ^63^, ^99^, ^101^, ^102^ ^]^ Since the latter is related to the molecular quadrupole, the magnitude of energy level bending at the interface correlates with the molecular quadrupole moment. As a rule of thumb, $Q_{\pi }\approx 100\;ea_{0}^{2}$ (75 Debye Å) provides a balance between efficient exciton dissociation and open circuit voltage losses.^[^ ^63^ ^]^

**Figure 7:**
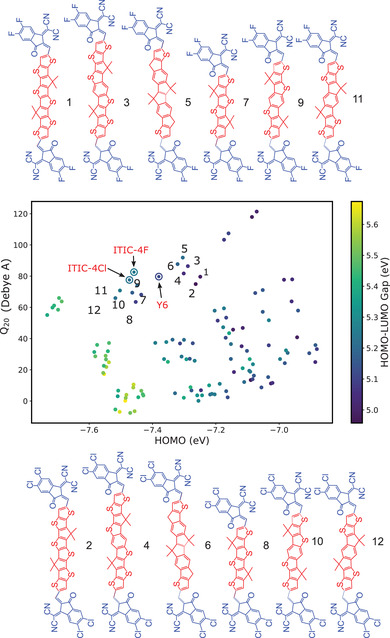
The *Q*_20_‐HOMO plot for 121 A–D–A compounds and high‐performance NFAs (ITIC‐4F, ITIC‐4Cl and Y6). Each scatter is colored according to its corresponding HOMO‐LUMO gap value.

Such molecular design rules have been used recently to pre‐screen a computer‐generated database of 121 compounds.^[^ ^63^ ^]^ As a result, 12 potential candidates, shown in **Figure** **7**, could be identified. Eight of these compounds have already been synthesized, resulting in 10% to 15% efficient solar cells, confirming the practicality of the proposed prescreening.

Conjugated bridges between the donor and the acceptor blocks, as well as side groups offer an extra degree of freedom for the NFA optimization. Conjugated bridges, for example, increase the conjugation length of NFAs, leading to a deeper LUMO, a shallower HOMO and a smaller optical gap. These properties are closely related to the device characteristics, such as open circuit voltage, internal quantum efficiency, and light absorption.^[^ ^63^ ^]^ The side groups can be used to tune the solubility and crystal structure of NFAs.^[^ ^103^ ^]^

## 4 Nanographenes

In addition to the aforementioned examples, several other types of conjugated organics molecules emerged recently: polycyclic aromatic hydrocarbons (PAH), nanographenes (NG), and graphene nanoribbons (GNRs).^[^ ^2^, ^3^, ^104^, ^105^, ^106^ ^]^ Tunable optical gap—literally starting from zero eV for graphene—high electronic mobilities,^[^ ^107^ ^]^ mechanical strength,^[^ ^108^ ^]^ and thermal conductivity^[^ ^109^ ^]^ make them interesting for electronic applications such as electrodes, sensors, and field effect transistors.^[^ ^110^ ^]^

**Figure 8:**
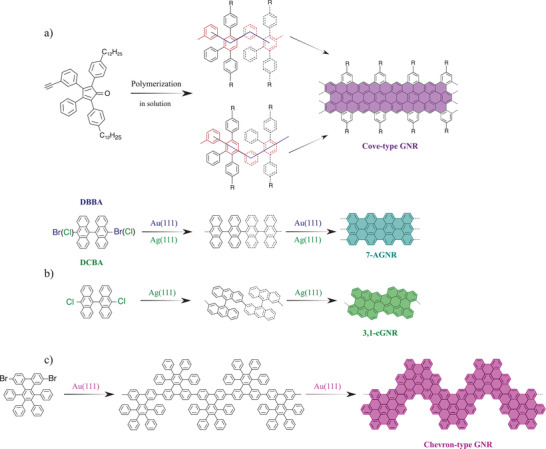
Example of molecular precursors and resulting GNRs after in‐solution or thermally activated on‐surface synthesis. Molecular precursors are responsible for the width and edge structure of the ribbons, providing control over the band gap by design. a) GNRs synthesized in solution via Diels–Alder polymerization of small nonsymmetrical monomers, which induces positional isomerism (R=C_12_H_25_).^[^ ^111^, ^112^ ^]^ b) Combination of precursor molecules and metal‐surfaces result in the same 7‐armchair‐type GNR (7‐AGNR). 10,10′‐dichloro‐9,9′‐bianthryl (DCBA) on Ag(111)^[^ ^113^ ^]^ and 10,10′‐dibromo‐9,9′‐bianthryl (DBBA) on Au(111).^[^ ^114^ ^]^ The chiral GNR (3,1‐cAGNR) was also obtained by DCBA on Ag(111). c) Chevron‐like AGNRs.^[^ ^114^, ^115^ ^]^

GNRs' optical properties, and their semi‐conducting and metallic electronic structures depend on their chemical structure, width and edge configuration.^[^ ^3^, ^115^, ^116^, ^117^, ^118^ ^]^ Potential applications of GNRs in fields such as nano and optoelectronics, photonics, and quantum computing motivate the search for new ways to synthesize them with well defined and perfectly controlled chemical structures. Top‐down techniques, such as “unzipping” carbon nanotubes, provide a straightforward method for GNRs production. However, this approach results in rough and chemically undefined edge shapes, which produce unpredictable electronic structures, compromising their application in optoelectronic devices. During the last years, bottom‐up chemical synthesis, either in solution^[^ ^3^, ^111^ ^]^ or surface assisted^[^ ^105^, ^110^, ^114^ ^]^ has been developed and provides atomically precise GNRs with tunable properties. Indeed, bottom‐up synthesis requires tailored molecular precursors, which after polymerization, graphitization, and planarization, produce atomically precise GNRs with specific edge shapes and widths. **Figure** **8** shows a few representative examples of how fine‐tuning of the electronic bandgap can be achieved through this structural perfection.^[^ ^111^, ^112^, ^114^, ^119^, ^120^, ^121^, ^122^, ^123^ ^]^

The current focus of computational methods is on single chains, such as polyphenylene precursors.^[^ ^112^ ^]^ Here, ab initio/ZINDO methods are used to predict excitation energies of short GNRs, molecular dynamics is employed to investigate side‐chain packing,^[^ ^124^ ^]^ and density functional theory is used to examine the effect of GNR width, edge geometry, and functional groups on the vibrational spectra of GNRs.^[^ ^125^ ^]^ More sophisticated methods, such as many‐body perturbation GW and Bethe Salpeter equation (BSE) approaches are used to calculate dielectric functions of GNRs and their polymer precursors,^[^ ^115^ ^]^ as well as their fundamental band gaps and Raman spectra.^[^ ^126^ ^]^

The rational design of these macromolecular materials so far has only found a few applications, but they find rapidly increasing attention: for example, GNR‐based hetero‐junctions^[^ ^127^ ^]^ have been reported. Doped with heteroatoms,^[^ ^128^ ^]^ nanographenes found their use in light emitting diodes as multiresonant thermally activated delayed fluorescent emitters.^[^ ^40^ ^]^ These compounds have very narrow‐band emission, high photoluminescence quantum yield, high chemical and thermal stabilities.^[^ ^41^ ^]^ Here a better theoretical understanding of the design rules is urgently needed, as well as the exploration of a wider chemical space.

## 5 Outlook

Overall, computational screening and testing has made significant progress over the last decades and is turning from a retroactive to a predictive tool. Nevertheless, a comprehensive methodology is still in far reach. It is clear that virtual screening relies on a well‐defined hierarchy of structure‐property relations or even more elaborate structure‐processing‐property relations. For polymers, for example, intermolecular and backbone conformation‐dependent contributions to the electronic properties depend on morphology and local packing, which in turn delicately rely on well defined and controlled sample preparation.

While precise structure‐property relations, for example molecular symmetry, can be readily used when generating the virtual database, the computationally demanding properties, such as solid state ionization energy or electron affinity, are useful for refining the database of potential structures. In fact, without the hierarchy of structure‐property relations, it is practically impossible to implement the high‐throughput screening scheme: the initial number of potential organic molecules is simply too big. In other words, a deep insight into the forward problem, that is predicting properties of interest from the chemical structure, is still required for designing a practical and useful prescreening procedure.

The prediction of material properties from molecular structures (forward problem) can be complemented by the machine learning models (MLs), which help to reduce the cost of quantum mechanical or multiscale simulations. The training set of ML can be based on experimentally available structures, such as organic molecules in the Cambridge Structural Database^[^ ^129^ ^]^ or generated by solving the forward problem for a set of computer‐generated structures.^[^ ^130^ ^]^ ML can also be used to propose structures with a given set of properties, addressing the inverse design problem.^[^ ^131^ ^]^ This process can be further optimized by using active machine learning^[^ ^132^, ^133^ ^]^ or generative models.^[^ ^134^ ^]^

## Conflict of Interest

The authors declare no conflict of interest.

## References

[1] M. D. Watson , A. Fechtenkötter , K. Müllen , Chem. Rev. 2001, 101, 1267. 11710221 10.1021/cr990322p

[2] X. Feng , W. Pisula , K. Müllen , Pure Appl. Chem. 2009, 81, 2203.

[3] A. Narita , X.‐Y. Wang , X. Feng , K. Müllen , Chem. Soc. Rev. 2015, 44, 6616. 26186682 10.1039/c5cs00183h

[4] C. K. Chiang , C. R. Fincher , Y. W. Park , A. J. Heeger , H. Shirakawa , E. J. Louis , S. C. Gau , A. G. MacDiarmid , Phys. Rev. Lett. 1977, 39, 1098.

[5] S. Chung , K. Cho , T. Lee , Adv. Sci. 2019, 6, 1801445. 10.1002/advs.201801445 PMC6425446 30937255

[6] A. J. Heeger , Rev. Mod. Phys. 2001, 73, 681.

[7] H. Shirakawa , Angew. Chem., Int. Ed. 2001, 40, 2574. 11458346

[8] A. G. MacDiarmid , Angew. Chem., Int. Ed. 2001, 40, 2581.

[9] A. J. Stone , *The Theory* of Intermolecular Forces, Clarendon Press, Oxford 1997. 10.1093/acprof:oso/9780199672394.001.0001

[10] C. Poelking , D. Andrienko , J. Chem. Theory Comput. 2016, 12, 4516. 27463038 10.1021/acs.jctc.6b00599

[11] P. Klüpfel , P. M. Dinh , P.‐G. Reinhard , E. Suraud , Phys. Rev. A 2013, 88, 052501.

[12] U. Salzner , R. Baer , J. Chem. Phys. 2009, 131, 231101. 20025305 10.1063/1.3269030

[13] H. Sun , S. Ryno , C. Zhong , M. K. Ravva , Z. Sun , T. Körzdörfer , J.‐L. Brédas , J. Chem. Theory Comput. 2016, 12, 2906. 27183355 10.1021/acs.jctc.6b00225

[14] T. Bereau , D. Andrienko , K. Kremer , APL Mater. 2016, 4, 053101.

[15] R. Olivares‐Amaya , C. Amador‐Bedolla , J. Hachmann , S. Atahan‐Evrenk , R. S. Sánchez‐Carrera , L. Vogt , A. Aspuru‐Guzik , Energy Environ. Sci. 2011, 4, 4849.

[16] P. Friederich , F. Häse , J. Proppe , A. Aspuru‐Guzik , Nat. Mater. 2021, 20, 750. 34045696 10.1038/s41563-020-0777-6

[17] D. Silver , A. Huang , C. J. Maddison , A. Guez , L. Sifre , G. van den Driessche , J. Schrittwieser , I. Antonoglou , V. Panneershelvam , M. Lanctot , S. Dieleman , D. Grewe , J. Nham , N. Kalchbrenner , I. Sutskever , T. Lillicrap , M. Leach , K. Kavukcuoglu , T. Graepel , D. Hassabis , Nature 2016, 529, 484. 26819042 10.1038/nature16961

[18] J. Jumper , R. Evans , A. Pritzel , T. Green , M. Figurnov , O. Ronneberger , K. Tunyasuvunakool , R. Bates , A. Žídek , A. Potapenko , A. Bridgland , C. Meyer , S. A. A. Kohl , A. J. Ballard , A. Cowie , B. Romera‐Paredes , S. Nikolov , R. Jain , J. Adler , T. Back , S. Petersen , D. Reiman , E. Clancy , M. Zielinski , M. Steinegger , M. Pacholska , T. Berghammer , S. Bodenstein , D. Silver , O. Vinyals , et al., Nature 2021, 596, 583. 34265844 10.1038/s41586-021-03819-2 PMC8371605

[19] J. Kirkpatrick , B. McMorrow , D. H. P. Turban , A. L. Gaunt , J. S. Spencer , A. G. D. G. Matthews , A. Obika , L. Thiry , M. Fortunato , D. Pfau , L. R. Castellanos , S. Petersen , A. W. R. Nelson , P. Kohli , P. Mori‐Sánchez , D. Hassabis , A. J. Cohen , Science 2021, 374, 1385. 34882476 10.1126/science.abj6511

[20] P. Kordt , J. J. Van Der Holst , M. Al Helwi , W. Kowalsky , F. May , A. Badinski , C. Lennartz , D. Andrienko , Adv. Funct. Mater. 2015, 25, 1955.

[21] L. Paterson , F. May , D. Andrienko , J. Appl. Phys. 2020, 128, 160901.

[22] G. Hong , X. Gan , C. Leonhardt , Z. Zhang , J. Seibert , J. M. Busch , S. Bräse , Adv. Mater. 2021, 33, 2005630. 10.1002/adma.202005630 33458866

[23] X. Ai , E. W. Evans , S. Dong , A. J. Gillett , H. Guo , Y. Chen , T. J. Hele , R. H. Friend , F. Li , Nature 2018, 563, 536. 30464267 10.1038/s41586-018-0695-9

[24] Q. Wei , N. Fei , A. Islam , T. Lei , L. Hong , R. Peng , X. Fan , L. Chen , P. Gao , Z. Ge , Adv. Opt. Mater. 2018, 6, 1800512.

[25] H. Guo , Q. Peng , X. K. Chen , Q. Gu , S. Dong , E. W. Evans , A. J. Gillett , X. Ai , M. Zhang , D. Credgington , V. Coropceanu , R. H. Friend , J. L. Bredas , F. Li , Nat. Mater. 2019, 18, 977. 31332338 10.1038/s41563-019-0433-1

[26] L. Paterson , A. Mondal , P. Heimel , R. Lovrincic , F. May , C. Lennartz , D. Andrienko , Adv. Electron. Mater. 2019, 5, 1900646.

[27] H. Uoyama , K. Goushi , K. Shizu , H. Nomura , C. Adachi , Nature 2012, 492, 234. 23235877 10.1038/nature11687

[28] J. Eng , T. J. Penfold , Commun. Chem. 2021, 4, 21. 10.1038/s42004-021-00533-y PMC9814861 36697585

[29] K. Zhang , J. Liu , Y. Zhang , J. Fan , C. K. Wang , L. Lin , J. Phys. Chem. C 2019, 123, 40.

[30] B. Minaev , G. Baryshnikov , H. Agren , Phys. Chem. Chem. Phys. 2014, 16, 1719. 24346346 10.1039/c3cp53806k

[31] N. Aizawa , Y. Harabuchi , S. Maeda , Y.‐J. Pu , Nat. Commun. 2020, 11, 3909. 32764588 10.1038/s41467-020-17777-2 PMC7411052

[32] Y. Niu , Q. Peng , C. Deng , X. Gao , Z. Shuai , J. Phys. Chem. A 2010, 114, 7817. 20666533 10.1021/jp101568f

[33] X.‐K. Chen , B. W. Bakr , M. Auffray , Y. Tsuchiya , C. D. Sherrill , C. Adachi , J.‐L. Bredas , J. Phys. Chem. Lett. 2019, 10, 3260. 31141375 10.1021/acs.jpclett.9b01220

[34] S. Weissenseel , N. A. Drigo , L. G. Kudriashova , M. Schmid , T. Morgenstern , K.‐H. Lin , A. Prlj , C. Corminboeuf , A. Sperlich , W. Brütting , M. K. Nazeeruddin , V. Dyakonov , J. Phys. Chem. C 2019, 123, 27778.

[35] K. Stavrou , L. G. Franca , A. P. Monkman , ACS Appl. Electron. Mater. 2020, 2, 2868. 32984822 10.1021/acsaelm.0c00514 PMC7513578

[36] X.‐Q. Wang , S.‐Y. Yang , Q.‐S. Tian , C. Zhong , Y.‐K. Qu , Y.‐J. Yu , Z.‐Q. Jiang , L.‐S. Liao , Angew. Chem., Int. Ed. 2021, 60, 5213. 10.1002/anie.202011384 33225601

[37] X. Tang , L. S. Cui , H. C. Li , A. J. Gillett , F. Auras , Y. K. Qu , C. Zhong , S. T. Jones , Z. Q. Jiang , R. H. Friend , L. S. Liao , Nat. Mater. 2020, 19, 1332. 32541938 10.1038/s41563-020-0710-z

[38] K.‐L. Woon , C.‐L. Yi , K.‐C. Pan , M. K. Etherington , C.‐C. Wu , K.‐T. Wong , A. P. Monkman , J. Phys. Chem. C 2019, 123, 12400. 10.1021/acs.jpcc.9b01900 PMC7493288 32952765

[39] P. Rajamalli , F. Rizzi , W. Li , M. A. Jinks , A. K. Gupta , B. A. Laidlaw , I. D. Samuel , T. J. Penfold , S. M. Goldup , E. Zysman‐Colman , Angew. Chem., Int. Ed. 2021, 60, 12066. 10.1002/anie.202101870 PMC8251797 33666324

[40] H. Hirai , K. Nakajima , S. Nakatsuka , K. Shiren , J. Ni , S. Nomura , T. Ikuta , T. Hatakeyama , Angew. Chem., Int. Ed. 2015, 54, 13581. 10.1002/anie.201506335 26380959

[41] S. Madayanad Suresh , D. Hall , D. Beljonne , Y. Olivier , E. Zysman‐Colman , Adv. Funct. Mater. 2020, 30, 1908677.

[42] A. Pershin , D. Hall , V. Lemaur , J. C. Sancho‐Garcia , L. Muccioli , E. Zysman‐Colman , D. Beljonne , Y. Olivier , Nat. Commun. 2019, 10, 3. 30723203 10.1038/s41467-019-08495-5 PMC6363735

[43] D. Hall , K. Stavrou , E. Duda , A. Danos , S. Bagnich , S. Warriner , A. M. Z. Slawin , D. Beljonne , A. Köhler , A. Monkman , Y. Olivier , E. Zysman‐Colman , Mater. Horiz. 2022, 9, 1068. 35067689 10.1039/d1mh01383a

[44] D. Hall , J. C. Sancho‐garcia , A. Pershin , D. Beljonne , E. Zysman , ChemRxiv 2021. 10.33774/chemrxiv-2021-496gn

[45] J. C. Sancho‐García , E. Brémond , G. Ricci , A. J. Pérez‐Jiménez , Y. Olivier , C. Adamo , J. Chem. Phys. 2022, 156, 034105. 35065561 10.1063/5.0076545

[46] J. Ehrmaier , E. J. Rabe , S. R. Pristash , K. L. Corp , C. W. Schlenker , A. L. Sobolewski , W. Domcke , J. Phys. Chem. A 2019, 8099. 31466450 10.1021/acs.jpca.9b06215

[47] P. De Silva , C. A. Kim , T. Zhu , T. Van Voorhis , Chem. Mater. 2019, 31, 6995.

[48] A. L. Sobolewski , W. Domcke , J. Phys. Chem. Lett. 2021, 12, 6852. 34279950 10.1021/acs.jpclett.1c01926

[49] N. B. Kotadiya , P. W. Blom , G. J. A. Wetzelaer , Nat. Photonics 2019, 13, 765.

[50] N. B. Kotadiya , H. Lu , A. Mondal , Y. Ie , D. Andrienko , P. W. Blom , G. J. A. Wetzelaer , Nat. Mater. 2018, 17, 329. 29459747 10.1038/s41563-018-0022-8

[51] N. B. Kotadiya , A. Mondal , P. W. Blom , D. Andrienko , G. J. A. Wetzelaer , Nat. Mater. 2019, 18, 1182. 31548633 10.1038/s41563-019-0473-6

[52] W. Liu , N. B. Kotadiya , P. W. M. Blom , G. A. H. Wetzelaer , D. Andrienko , Adv. Mater. Technol. 2021, 6, 2000120.

[53] K.‐H. Lin , A. Prlj , L. Yao , N. Drigo , H.‐H. Cho , M. K. Nazeeruddin , K. Sivula , C. Corminboeuf , Chem. Mater 2019, 31, 6605.

[54] K.‐H. Lin , A. Prlj , C. Corminboeuf , J. Mater. Chem. C 2018, 6, 960.

[55] A. Mondal , L. Paterson , J. Cho , K.‐H. Lin , B. van der Zee , G.‐J. A. H. Wetzelaer , A. Stankevych , A. Vakhnin , J.‐J. Kim , A. Kadashchuk , P. W. M. Blom , F. May , D. Andrienko , Chem. Phys. Rev. 2021, 2, 031304.

[56] K.‐H. Lin , G.‐J. A. H. Wetzelaer , P. W. M. Blom , D. Andrienko , Front. Chem. 2021, 9, 800027. 34976956 10.3389/fchem.2021.800027 PMC8716429

[57] P. Jiang , J. Miao , X. Cao , H. Xia , K. Pan , T. Hua , X. Lv , Z. Huang , Y. Zou , C. Yang , Adv. Mater. 2021, 34, 2106954. 10.1002/adma.202106954 34766672

[58] R. Fitzner , E. Reinold , A. Mishra , E. Mena‐Osteritz , H. Ziehlke , C. Körner , K. Leo , M. Riede , M. Weil , O. Tsaryova , A. Weiß , C. Uhrich , M. Pfeiffer , P. Bäuerle , Adv. Funct. Mater. 2011, 21, 897.

[59] S. Holliday , Y. Li , C. K. Luscombe , Prog. Polym. Sci. 2017, 70, 34.

[60] L. Benatto , C. Marchiori , T. Talka , M. Aramini , N. Yamamoto , S. Huotari , L. Roman , M. Koehler , Thin Solid Films 2020, 697, 137827.

[61] J. C. Hummelen , B. W. Knight , F. LePeq , F. Wudl , J. Yao , C. L. Wilkins , J. Org. Chem. 1995, 60, 532.

[62] N. S. Sariciftci , L. Smilowitz , A. J. Heeger , F. Wudl , Science 1992, 258, 1474. 17755110 10.1126/science.258.5087.1474

[63] A. Markina , K.‐H. Lin , W. Liu , C. Poelking , Y. Firdaus , D. R. Villalva , J. I. Khan , S. H. K. Paleti , G. T. Harrison , J. Gorenflot , W. Zhang , S. De Wolf , I. McCulloch , T. D. Anthopoulos , D. Baran , F. Laquai , D. Andrienko , Adv. Energy Mater. 2021, 11, 2102363.

[64] J. Zhao , Y. Li , G. Yang , K. Jiang , H. Lin , H. Ade , W. Ma , H. Yan , Nat. Energy 2016, 1, 15027.

[65] D. Qian , Z. Zheng , H. Yao , W. Tress , T. R. Hopper , S. Chen , S. Li , J. Liu , S. Chen , J. Zhang , X.‐K. Liu , B. Gao , L. Ouyang , Y. Jin , G. Pozina , I. A. Buyanova , W. M. Chen , O. Inganäs , V. Coropceanu , J.‐L. Bredas , H. Yan , J. Hou , F. Zhang , A. A. Bakulin , F. Gao , Nat. Mater. 2018, 17, 703. 30013057 10.1038/s41563-018-0128-z

[66] C. Yan , S. Barlow , Z. Wang , H. Yan , A. K.‐Y. Jen , S. R. Marder , X. Zhan , Nat. Rev. Mater. 2018, 3, 18003.

[67] A. Armin , W. Li , O. J. Sandberg , Z. Xiao , L. Ding , J. Nelson , D. Neher , K. Vandewal , S. Shoaee , T. Wang , H. Ade , T. Heumüller , C. Brabec , P. Meredith , Adv. Energy Mater. 2021, 11, 2003570.

[68] P. Meredith , W. Li , A. Armin , Adv. Energy Mater. 2020, 10, 2001788.

[69] Y. Cui , H. Yao , J. Zhang , T. Zhang , Y. Wang , L. Hong , K. Xian , B. Xu , S. Zhang , J. Peng , Z. Wei , F. Gao , J. Hou , Nat. Commun. 2019, 10, 2515. 31175276 10.1038/s41467-019-10351-5 PMC6555805

[70] X. Xu , K. Feng , Z. Bi , W. Ma , G. Zhang , Q. Peng , Adv. Mater. 2019, 31, 1901872. 10.1002/adma.201901872 31157474

[71] Y. Lin , Y. Firdaus , M. I. Nugraha , F. Liu , S. Karuthedath , A.‐H. Emwas , W. Zhang , A. Seitkhan , M. Neophytou , H. Faber , E. Yengel , I. McCulloch , L. Tsetseris , F. Laquai , T. D. Anthopoulos , Adv. Sci. 2020, 7, 1903419. 10.1002/advs.201903419 PMC7141031 32274320

[72] Q. Liu , Y. Jiang , K. Jin , J. Qin , J. Xu , W. Li , J. Xiong , J. Liu , Z. Xiao , K. Sun , S. Yang , X. Zhang , L. Ding , Sci. Bull. 2020, 65, 272. 10.1016/j.scib.2020.01.001 36659090

[73] L. Meng , Y. Zhang , X. Wan , C. Li , X. Zhang , Y. Wang , X. Ke , Z. Xiao , L. Ding , R. Xia , H.‐L. Yip , Y. Cao , Y. Chen , Science 2018, 361, 1094. 30093603 10.1126/science.aat2612

[74] M. B. Salim , R. Nekovei , R. Jeyakumar , Sol. Energy 2020, 198, 160.

[75] S. D. Dimitrov , A. A. Bakulin , C. B. Nielsen , B. C. Schroeder , J. Du , H. Bronstein , I. McCulloch , R. H. Friend , J. R. Durrant , J. Am. Chem. Soc. 2012, 134, 18189. 23094985 10.1021/ja308177d

[76] K. H. Hendriks , A. S. G. Wijpkema , J. J. van Franeker , M. M. Wienk , R. A. J. Janssen , J. Am. Chem. Soc. 2016, 138, 10026. 27452683 10.1021/jacs.6b05868

[77] S. Karuthedath , J. Gorenflot , Y. Firdaus , N. Chaturvedi , C. S. P. De Castro , G. T. Harrison , J. I. Khan , A. Markina , A. H. Balawi , T. A. D. Peña , W. Liu , R.‐Z. Liang , A. Sharma , S. H. K. Paleti , W. Zhang , Y. Lin , E. Alarousu , D. H. Anjum , P. M. Beaujuge , S. De Wolf , I. McCulloch , T. D. Anthopoulos , D. Baran , D. Andrienko , F. Laquai , Nat. Mater. 2021, 20, 378. 33106652 10.1038/s41563-020-00835-x

[78] X. Shen , G. Han , D. Fan , Y. Xie , Y. Yi , J. Phys. Chem. C 2015, 119, 11320.

[79] S. Shoaee , S. Subramaniyan , H. Xin , C. Keiderling , P. S. Tuladhar , F. Jamieson , S. A. Jenekhe , J. R. Durrant , Adv. Funct. Mater. 2013, 23, 3286.

[80] T. M. Burke , S. Sweetnam , K. Vandewal , M. D. McGehee , Adv. Energy Mater. 2015, 5, 1500123.

[81] K. Vandewal , S. Albrecht , E. T. Hoke , K. R. Graham , J. Widmer , J. D. Douglas , M. Schubert , W. R. Mateker , J. T. Bloking , G. F. Burkhard , A. Sellinger , J. M. J. Fréchet , A. Amassian , M. K. Riede , M. D. McGehee , D. Neher , A. Salleo , Nat. Mater. 2014, 13, 63. 24240240 10.1038/nmat3807

[82] J. Benduhn , K. Tvingstedt , F. Piersimoni , S. Ullbrich , Y. Fan , M. Tropiano , K. A. McGarry , O. Zeika , M. K. Riede , C. J. Douglas , S. Barlow , S. R. Marder , D. Neher , D. Spoltore , K. Vandewal , Nat. Energy 2017, 2, 17053.

[83] K. Nakano , Y. Chen , B. Xiao , W. Han , J. Huang , H. Yoshida , E. Zhou , K. Tajima , Nat. Commun. 2019, 10, 2520. 31175294 10.1038/s41467-019-10434-3 PMC6555791

[84] J. Benduhn , F. Piersimoni , G. Londi , A. Kirch , J. Widmer , C. Koerner , D. Beljonne , D. Neher , D. Spoltore , K. Vandewal , Adv. Energy Mater. 2018, 8, 1800451.

[85] K. Vandewal , S. Mertens , J. Benduhn , Q. Liu , J. Phys. Chem. Lett. 2020, 11, 129. 31829597 10.1021/acs.jpclett.9b02719

[86] G. Sini , M. Schubert , C. Risko , S. Roland , O. P. Lee , Z. Chen , T. V. Richter , D. Dolfen , V. Coropceanu , S. Ludwigs , U. Scherf , A. Facchetti , J. M. J. Fréchet , D. Neher , Adv. Energy Mater. 2018, 8, 1702232.

[87] S. Albrecht , K. Vandewal , J. R. Tumbleston , F. S. U. Fischer , J. D. Douglas , J. M. J. Fréchet , S. Ludwigs , H. Ade , A. Salleo , D. Neher , Adv. Mater. 2014, 26, 2533. 24574091 10.1002/adma.201305283

[88] D. Bartesaghi , I. d. C. Pérez , J. Kniepert , S. Roland , M. Turbiez , D. Neher , L. J. A. Koster , Nat. Commun. 2015, 6, 7083. 25947637 10.1038/ncomms8083 PMC4432638

[89] M. L. Tietze , W. Tress , S. Pfützner , C. Schünemann , L. Burtone , M. Riede , K. Leo , K. Vandewal , S. Olthof , P. Schulz , A. Kahn , Phys. Rev. B 2013, 88, 085119.

[90] K. R. Graham , G. O. N. Ndjawa , S. M. Conron , R. Munir , K. Vandewal , J. J. Chen , S. Sweetnam , M. E. Thompson , A. Salleo , M. D. McGehee , A. Amassian , Adv. Energy Mater. 2016, 6, 1601211.

[91] J. Hou , O. Inganäs , R. H. Friend , F. Gao , Nat. Mater. 2018, 17, 119. 29358765 10.1038/nmat5063

[92] M. A. Alamoudi , J. I. Khan , Y. Firdaus , K. Wang , D. Andrienko , P. M. Beaujuge , F. Laquai , ACS Energy Lett. 2018, 3, 802.

[93] M. C. Scharber , N. S. Sariciftci , Prog. Polym. Sci. 2013, 38, 1929. 24302787 10.1016/j.progpolymsci.2013.05.001 PMC3837184

[94] D. Neher , J. Kniepert , A. Elimelech , L. J. A. Koster , Sci. Rep. 2016, 6, 24861. 27112905 10.1038/srep24861 PMC4845057

[95] D. Baran , R. S. Ashraf , D. A. Hanifi , M. Abdelsamie , N. Gasparini , J. A. Röhr , S. Holliday , A. Wadsworth , S. Lockett , M. Neophytou , C. J. M. Emmott , J. Nelson , C. J. Brabec , A. Amassian , A. Salleo , T. Kirchartz , J. R. Durrant , I. McCulloch , Nat. Mater. 2017, 16, 363. 27869824 10.1038/nmat4797

[96] Y. Firdaus , V. M. Le Corre , S. Karuthedath , W. Liu , A. Markina , W. Huang , S. Chattopadhyay , M. M. Nahid , M. I. Nugraha , Y. Lin , A. Seitkhan , A. Basu , W. Zhang , I. McCulloch , H. Ade , J. Labram , F. Laquai , D. Andrienko , L. J. A. Koster , T. D. Anthopoulos , Nat. Commun. 2020, 11, 5220. 33060574 10.1038/s41467-020-19029-9 PMC7562871

[97] A. F. Paterson , R. Li , A. Markina , L. Tsetseris , S. MacPhee , H. Faber , A.‐H. Emwas , J. Panidi , H. Bristow , A. Wadsworth , D. Baran , D. Andrienko , M. Heeney , I. McCulloch , T. D. Anthopoulos , J. Mater. Chem. C 2021, 9, 4486.

[98] C. Poelking , M. Tietze , C. Elschner , S. Olthof , D. Hertel , B. Baumeier , F. Würthner , K. Meerholz , K. Leo , D. Andrienko , Nat. Mater. 2015, 14, 434. 25532071 10.1038/nmat4167

[99] M. Schwarze , K. S. Schellhammer , K. Ortstein , J. Benduhn , C. Gaul , A. Hinderhofer , L. Perdigón Toro , R. Scholz , J. Kublitski , S. Roland , M. Lau , C. Poelking , D. Andrienko , G. Cuniberti , F. Schreiber , D. Neher , K. Vandewal , F. Ortmann , K. Leo , Nat. Commun. 2019, 10, 2466. 31165738 10.1038/s41467-019-10435-2 PMC6549189

[100] L. Perdigón‐Toro , H. Zhang , A. Markina , J. Yuan , S. M. Hosseini , C. M. Wolff , G. Zuo , M. Stolterfoht , Y. Zou , F. Gao , D. Andrienko , S. Shoaee , D. Neher , Adv. Mater. 2020, 32, 1906763. 10.1002/adma.201906763 31975446

[101] G. D'Avino , L. Muccioli , F. Castet , C. Poelking , D. Andrienko , Z. G. Soos , J. Cornil , D. Beljonne , J. Phys.: Condens. Matter 2016, 28, 433002. 27603960 10.1088/0953-8984/28/43/433002

[102] M. Schwarze , W. Tress , B. Beyer , F. Gao , R. Scholz , C. Poelking , K. Ortstein , A. A. Günther , D. Kasemann , D. Andrienko , K. Leo , Science 2016, 352, 1446. 27313043 10.1126/science.aaf0590

[103] F. Shen , J. Xu , X. Li , C. Zhan , J. Mater. Chem. A 2018, 6, 15433.

[104] S. Dutta , S. K. Pati , J. Mater. Chem. 2010, 20, 8207.

[105] H. Wang , H. S. Wang , C. Ma , L. Chen , C. Jiang , C. Chen , X. Xie , A.‐P. Li , X. Wang , Nat. Rev. Phys. 2021, 3, 791.

[106] M. C. Drummer , V. Singh , N. Gupta , J. L. Gesiorski , R. B. Weerasooriya , K. D. Glusac , Photosynth. Res. 2022, 151, 163. 33963981 10.1007/s11120-021-00838-y

[107] K. I. Bolotin , K. J. Sikes , Z. Jiang , M. Klima , G. Fudenberg , J. Hone , P. Kim , H. L. Stormer , Solid State Commun. 2008, 146, 351.

[108] C. Lee , X. Wei , J. W. Kysar , J. Hone , Science 2008, 321, 385. 18635798 10.1126/science.1157996

[109] E. Pop , V. Varshney , A. K. Roy , MRS Bull. 2012, 37, 1273.

[110] Z. Chen , A. Narita , K. Müllen , Adv. Mater. 2020, 32, 2001893. 10.1002/adma.202001893 32945038

[111] A. Narita , X. Feng , Y. Hernandez , S. A. Jensen , M. Bonn , H. Yang , I. A. Verzhbitskiy , C. Casiraghi , M. R. Hansen , A. H. R. Koch , G. Fytas , O. Ivasenko , B. Li , K. S. Mali , T. Balandina , S. Mahesh , S. De Feyter , K. Müllen , Nat. Chem. 2014, 6, 126. 24451588 10.1038/nchem.1819

[112] N. C. Forero‐Martinez , B. Baumeier , K. Kremer , Macromolecules 2019, 52, 5307. 31543550 10.1021/acs.macromol.9b00819 PMC6750833

[113] P. H. Jacobse , K. A. Simonov , M. J. J. Mangnus , G. I. Svirskiy , A. V. Generalov , A. S. Vinogradov , A. Sandell , N. Mårtensson , A. B. Preobrajenski , I. Swart , J. Phys. Chem. C 2019, 123, 8892. 10.1021/acs.jpcc.8b12209 PMC6463537 31001369

[114] J. Cai , P. Ruffieux , R. Jaafar , M. Bieri , T. Braun , S. Blankenburg , M. Muoth , A. P. Seitsonen , M. Saleh , X. Feng , K. Müllen , R. Fasel , Nature 2010, 466, 470. 20651687 10.1038/nature09211

[115] R. Denk , A. Lodi‐Rizzini , S. Wang , M. Hohage , P. Zeppenfeld , J. Cai , R. Fasel , P. Ruffieux , R. F. J. Berger , Z. Chen , A. Narita , X. Feng , K. Müllen , R. Biagi , V. D. Renzi , D. Prezzi , A. Ruini , A. Ferretti , Nanoscale 2017, 9, 18326. 29143040 10.1039/c7nr06175g

[116] L. Talirz , P. Ruffieux , R. Fasel , Adv. Mater. 2016, 28, 6222. 26867990 10.1002/adma.201505738

[117] A. Narita , Z. Chen , Q. Chen , K. Müllen , Chem. Sci. 2019, 10, 964. 30774890 10.1039/c8sc03780a PMC6349060

[118] X. Yu , S. Fu , M. Mandal , X. Yao , Z. Liu , W. Zheng , P. Samorì , A. Narita , K. Müllen , D. Andrienko , M. Bonn , H. I. Wang , The Journal of Chemical Physics 2022, 13, 892. 10.1063/5.0081074 35183096

[119] P. Han , K. Akagi , F. Federici Canova , H. Mutoh , S. Shiraki , K. Iwaya , P. S. Weiss , N. Asao , T. Hitosugi , ACS Nano 2014, 8, 9181. 25162921 10.1021/nn5028642

[120] C. Sánchez‐Sánchez , T. Dienel , O. Deniz , P. Ruffieux , R. Berger , X. Feng , K. Müllen , R. Fasel , ACS Nano 2016, 10, 8006. 27428831 10.1021/acsnano.6b04025

[121] I. C.‐Y. Hou , A. Narita , K. Müllen , Macromol. Chem. Phys. 2020, 221, 1900374.

[122] M. G. Schwab , A. Narita , Y. Hernandez , T. Balandina , K. S. Mali , S. De Feyter , X. Feng , K. Müllen , J. Am. Chem. Soc. 2012, 134, 18169. 23082776 10.1021/ja307697j

[123] A. Baun , Z. Wang , S. Morsbach , Z. Qiu , A. Narita , G. Fytas , K. Müllen , Macromolecules 2020, 53, 5756. 32742022 10.1021/acs.macromol.0c00810 PMC7392475

[124] Y. Fogel , L. Zhi , A. Rouhanipour , D. Andrienko , H. J. Räder , K. Müllen , Macromolecules 2009, 42, 6878.

[125] I. A. Verzhbitskiy , M. D. Corato , A. Ruini , E. Molinari , A. Narita , Y. Hu , M. G. Schwab , M. Bruna , D. Yoon , S. Milana , X. Feng , K. Müllen , A. C. Ferrari , C. Casiraghi , D. Prezzi , Nano Lett. 2016, 16, 3442. 26907096 10.1021/acs.nanolett.5b04183 PMC4901367

[126] L. Talirz , H. Söde , T. Dumslaff , S. Wang , J. R. Sanchez‐Valencia , J. Liu , P. Shinde , C. A. Pignedoli , L. Liang , V. Meunier , N. C. Plumb , M. Shi , X. Feng , A. Narita , K. Müllen , R. Fasel , P. Ruffieux , ACS Nano 2017, 11, 1380. 28129507 10.1021/acsnano.6b06405

[127] J. Cai , C. A. Pignedoli , L. Talirz , P. Ruffieux , H. Söde , L. Liang , V. Meunier , R. Berger , R. Li , X. Feng , K. Müllen , R. Fasel , Nat. Nanotechnol. 2014, 9, 896. 25194948 10.1038/nnano.2014.184

[128] X.‐Y. Wang , X. Yao , A. Narita , K. Müllen , Acc. Chem. Res. 2019, 52, 2491. 31478641 10.1021/acs.accounts.9b00322 PMC6751794

[129] Ö. H. Omar , T. Nematiaram , A. Troisi , D. Padula , Sci. Data 2022, 9, 54. 35165288 10.1038/s41597-022-01142-7 PMC8844419

[130] H. S. Kwak , Y. An , D. J. Giesen , T. F. Hughes , C. T. Brown , K. Leswing , H. Abroshan , M. D. Halls , Front. Chem. 2022, 9, 800370. 35111730 10.3389/fchem.2021.800370 PMC8802168

[131] K. Kim , S. Kang , J. Yoo , Y. Kwon , Y. Nam , D. Lee , I. Kim , Y.‐S. Choi , Y. Jung , S. Kim , W.‐J. Son , J. Son , H. S. Lee , S. Kim , J. Shin , S. Hwang , npj Comput. Mater. 2018, 4, 67.

[132] H. Abroshan , H. S. Kwak , Y. An , C. Brown , A. Chandrasekaran , P. Winget , M. D. Halls , Front. Chem. 2022, 9, 800371. 35111731 10.3389/fchem.2021.800371 PMC8802167

[133] B. Mohr , K. Shmilovich , I. S Kleinwächter , D. Schneider , A. L. Ferguson , T. Bereau , Chem. Sci. 2022. 10.1039/d2sc00116k. PMC9019913 35656132

[134] C. Kunkel , J. T. Margraf , K. Chen , H. Oberhofer , K. Reuter , Nature Communications 2021, 12, 2422. 10.1038/s41467-021-22611-4 PMC8065160 33893287

